# 

**Published:** 1907-04-15

**Authors:** 


### CONTENTS

Event and Comment - - - - - - - - - - 163

Why the Profession should adopt Porcelain as a Standard Filling Material, - - - - - *By W. T. Reeves, D.D.S.* 166

Incidents in Porcelain Work, - - *By Dr. J. M. Thompson* 177

The Treatment of Sensitive Dentin, with Special Reference to the Production of Anesthesia by Pressure, *By W. D. Miller, M.D., D.D.S.* 187

With Our Editors - - - - - - - - - 194

Bibliography - - - - - - - - - - 201

Indents - - - - - - - - - - - 204

Announcements - - - - - - - - - - 215

